# Primary vaccination with the 10-valent pneumococcal non-typeable *Haemophilus influenzae *protein D conjugate vaccine (PHiD-CV) in infants in Mali and Nigeria: a randomized controlled trial

**DOI:** 10.1186/1471-2458-11-882

**Published:** 2011-11-23

**Authors:** Alassane Dicko, Olumuyiwa O Odusanya, Abdoulbaki I Diallo, Gaoussou Santara, Amadou Barry, Amagana Dolo, Aminata Diallo, Yetunde A Kuyinu, Omolara A Kehinde, Nancy François, Dorota Borys, Juan P Yarzabal, Marta Moreira, Lode Schuerman

**Affiliations:** 1 Malaria Research and Training Center, Faculty of Medicine, Pharmacy and Dentistry, University of Bamako, P.O. Box 1805, Bamako, Mali; 2 Department of Public Health, Faculty of Medicine, Pharmacy and Dentistry, University of Bamako, P.O. Box 1805, Bamako, Mali; 3 Department of Community Health and Primary Health Care, Lagos State University College of Medicine, P.M. B. 21266 Ikeja, Lagos, Nigeria; 4 Service de Pédiatrie, Centre Hospitalo-Universitaire Gabriel Touré, B.P. 267, Bamako, Mali; 5 Department of Pediatrics and Child Health, Lagos State University College of Medicine, P.M. B. 21266 Ikeja, Lagos, Nigeria; 6 GlaxoSmithKline Biologicals, Avenue Fleming 20, 1300 Wavre, Belgium

## Abstract

**Background:**

Pneumonia is still the leading cause of death among children in Africa, and pneumococcal serotypes 1 and 5 are frequently isolated from African children with invasive pneumococcal disease below the age of 5 years. The immunogenicity, safety and reactogenicity of 3-dose primary vaccination with the 10-valent pneumococcal non-typeable *Haemophilus influenzae *protein D conjugate vaccine (PHiD-CV) were evaluated in infants in Mali and Nigeria.

**Methods:**

In an open, randomized, controlled study, 357 infants received DTPw-HBV/Hib and OPV primary vaccination with (PHiD-CV group) or without (control group) PHiD-CV co-administration at 6, 10 and 14 weeks of age. Pneumococcal antibody responses and opsonophagocytic activity (OPA) were measured and adverse events (AEs) recorded.

**Results:**

One month post-dose 3, ≥ 97.2% of PHiD-CV-vaccinated infants had an antibody concentration ≥ 0.2 μg/mL for each vaccine pneumococcal serotype except for 6B (82.0%) and 23F (87.6%) versus < 10% in the control group except for serotypes 14 (35.7%) and 19F (22.5%). For each vaccine serotype, ≥ 93.3% of PHiD-CV recipients had an OPA titre ≥ 8, except for serotypes 1 (87.6%) and 6B (85.4%), compared to < 10% in the control group, except for serotypes 7F (42.9%), 9V (24.1%) and 14 (24.5%). Anti-protein D geometric mean antibody concentrations were 3791.8 and 85.4 EL.U/mL in the PHiD-CV and control groups, respectively. Overall incidences of solicited and unsolicited AEs were similar between groups.

**Conclusions:**

In sub-Saharan African infants, PHiD-CV was immunogenic for all vaccine pneumococcal serotypes and protein D. Vaccine tolerability was generally comparable between the PHiD-CV and control groups.

**Trial Registration:**

ClinicalTrials.gov identifier: NCT00678301.

## Background

In 2008, infectious diseases caused 68% of the estimated 8.8 million deaths in children younger than 5 years, with the largest percentage (18%) due to pneumonia [1]. Nearly half of pneumonia-related deaths in this age group were in sub-Saharan Africa. In 2008, in Mali and Nigeria alone, pneumonia caused almost 200,000 deaths in children below 5 years of age. Due to the high burden of childhood pneumonia in this region, donors such as the Global Alliance for Vaccines and Immunization (GAVI) support the introduction of pneumococcal conjugate vaccines in low-income African countries [2].

The contribution of *Streptococcus pneumoniae *to childhood pneumonia has been difficult to define given problems in establishing the aetiology of paediatric lower respiratory tract infection [3]. Studies that evaluated the efficacy of different pneumococcal conjugate vaccines against X-ray confirmed consolidated pneumonia in young children showed a 17% to 37% reduction, irrespective of aetiological agent [4-8]. Pneumococcal serotypes 1 and 5, which are not contained in the 7-valent pneumococcal CRM_197 _conjugate vaccine (7vCRM; *Prevenar/Prevnar™*, Pfizer Inc., New York, USA), are known to play an important role in childhood pneumococcal disease in Africa [9], where they are estimated to cause 22% of invasive pneumococcal disease (IPD) [10]. However, one study in 106 children with IPD in Mali reported over half (54%) of invasive disease cases were caused by serotype 5 [11].

The 10-valent pneumococcal non-typeable *Haemophilus influenzae *protein D conjugate vaccine (PHiD-CV; *Synflorix*™, GlaxoSmithKline [GSK] Biologicals, Rixensart, Belgium) contains pneumococcal serotypes 1, 5 and 7F in addition to the 7 serotypes included in 7vCRM (serotypes 4, 6B, 9V, 14, 18C, 19F, 23F). PHiD-CV also contains recombinant protein D as carrier protein for 8 of the 10 serotypes, which is derived from a cell surface lipoprotein of non-typeable *Haemophilus influenzae *(NTHi) that is highly conserved in both capsulated and non-capsulated strains [12-14]. PHiD-CV has been shown in studies conducted in Europe, Asia and Latin America to be immunogenic and well tolerated when administered in different primary vaccination schedules and when co-administered with other routine paediatric vaccines [15-22].

This is the first report of the assessment of PHiD-CV in an African population. We studied the immunogenicity, safety and reactogenicity of PHiD-CV when used for primary vaccination of infants in Mali and Nigeria according to the vaccination schedule at 6, 10 and 14 weeks of age, as used in the Expanded Program on Immunization (EPI) in both countries.

## Methods

### Study Objectives and Vaccines

The objectives of this phase III, randomized, open, controlled study were to assess the immunogenicity, safety and reactogenicity of 3-dose primary vaccination with PHiD-CV (*Synflorix*™) in sub-Saharan Africa. PHiD-CV contained 1 μg of each capsular polysaccharide for pneumococcal serotypes 1, 5, 6B, 7F, 9V, 14 and 23F, and 3 μg for serotype 4 conjugated individually to NTHi protein D, 3 μg of serotype 18C capsular polysaccharide conjugated to tetanus toxoid, and 3 μg of serotype 19F capsular polysaccharide conjugated to diphtheria toxoid. PHiD-CV was co-administered with combined diphtheria-tetanus-whole-cell pertussis-hepatitis B/*Haemophilus influenzae *type b (DTPw-HBV/Hib; *Zilbrix*™ Hib, GSK Biologicals, Rixensart, Belgium) and oral live attenuated poliovirus vaccines (OPV; *Polio Sabin*™, GSK Biologicals, Rixensart, Belgium). DTPw-HBV/Hib contained ≥ 30 IU diphtheria toxoid, ≥ 60 IU tetanus toxoid, ≥ 4 IU killed *Bordetella pertussis*, 10 μg recombinant hepatitis B surface antigen (HBs) and 2.5 μg Hib polysaccharide polyribosylribitol phosphate (PRP) conjugated to 5-10 μg tetanus toxoid. OPV contained 10^6 ^TCID_50 _poliovirus type 1, 10^5 ^TCID_50 _poliovirus type 2 and 10^5.5 ^TCID_50 _poliovirus type 3. PHiD-CV and DTPw-HBV/Hib were injected into the anterolateral region of the right and left thigh, respectively, and OPV was administered orally.

### Study Setting, Participants and Ethics

The study was conducted between June 2008 and December 2009 at 2 study sites. In Mali, the research team was based in the community health centre of the rural town of Oulessebougou and infants were recruited while visiting the local vaccination centre for their first DTPw-HBV/Hib dose. In Nigeria, the study was conducted in a purpose-built research suite at the Lagos State University Teaching Hospital, Ikeja, by the Department of Community Health and Primary Health Care. Infants were recruited from the daily immunization clinic and primary healthcare clinics around the hospital. Eligible subjects were healthy infants aged between 6 and 10 weeks at the time of first vaccination.

Written informed consent was obtained from a parent or legal guardian or the thumb print of an illiterate parent/guardian was obtained on the consent form, countersigned by an independent, literate witness. The trial was conducted according to Good Clinical Practice and the Declaration of Helsinki (Somerset West, 1996 version) and the protocol was reviewed and approved in Mali by the Ethical Committee of the Faculty of Medicine, Pharmacy and Dentistry of the University of Bamako and in Nigeria by the Health Research and Ethics Committee of the Lagos State University Teaching Hospital, Ikeja, Lagos. Authorization to conduct the study was obtained from the Ministry of Health in Mali and the National Agency for Food and Drug Administration and Control in Nigeria.

### Randomization

Infants were randomized (2:1 treatment allocation) to receive 3 doses of PHiD-CV co-administered with DTPw-HBV/Hib and OPV (PHiD-CV group) or 3 doses of DTPw-HBV/Hib and OPV without PHiD-CV co-administration (control group) at 6, 10 and 14 weeks of age. A randomization list generated at GSK Biologicals, Rixensart, using a standard SAS^® ^program was used to number the vaccines with a blocking scheme to ensure that balance between treatment groups was maintained. At the first vaccination visit, after verification of eligibility criteria, subjects received the vaccine dose with the lowest number still available at the study centre and the number used was recorded by the investigator.

### Immunogenicity Assessment

Blood samples were collected before vaccination and 1 month post-dose 3 and serum samples were stored at -20°C until analysis. GSK's 22F-inhibition enzyme-linked immunosorbent assay (22F-ELISA) with an assay cut-off of 0.05 μg/mL was used to measure anti-pneumococcal serotype-specific total IgG concentrations. Pneumococcal antibody responses were also analyzed using the threshold antibody concentration of 0.2 μg/mL, which is equivalent to the World Health Organization (WHO) recommended reference value of 0.35 μg/mL with the non-22F ELISA [23-25]. Opsonophagocytic activity (OPA) was measured on a random subset of 50% of the post-dose 3 samples (as planned in the protocol), using the OPA assay described previously, with a cut-off opsonic titre of 8 [26,27]. Results were presented as the reciprocal of dilution of serum (opsonic titre) able to sustain 50% killing of live pneumococci under the assay conditions. Antibodies against NTHi protein D were measured by a validated in-house ELISA with an assay cut-off of 100 ELISA units/mL (EL.U/mL). Serological responses against the co-administered DTPw-HBV/Hib vaccine were measured post-dose 3 in a subset of 50% of infants in the PHiD-CV group (those not tested for OPA as planned in the protocol) and all subjects in the control group. Validated ELISA assays were used with the following cut-off values: anti-PRP, 0.15 μg/mL; anti-diphtheria toxoid, 0.1 IU/mL; anti-tetanus toxoid, 0.1 IU/mL; anti-*B. pertussis*, 15 EL.U/mL; anti-HBs, 10 mIU/mL. Immune responses against the co-administered OPV vaccine were documented previously [28] and were therefore not assessed in our study.

### Safety Assessment

Local (pain, redness, swelling at the injection site) and general (fever, drowsiness, irritability, loss of appetite) symptoms were actively solicited for 4 days following each vaccine dose and were evaluated in all subjects using diary cards that were completed during daily home visits by study physicians (Mali) or study field workers (Nigeria). The field workers in Nigeria, who were selected from the community based on their willingness to participate, were trained on study procedures before and at regular intervals during the study.

Other adverse events (AEs) were recorded within a 31-day follow-up period after each vaccine dose and serious adverse events (SAEs), defined as any medical event resulting in death, any life-threatening event or any event causing disability, or requiring hospitalization or prolongation of hospitalization, were recorded during the entire study period up to 1 month after the third dose.

The intensity of each solicited AE was graded on a scale from 0 to 3. Pain at the injection site was considered to have a grade 3 intensity if the child cried when the limb was moved/was spontaneously painful, redness and swelling at the injection site if the diameter was > 30 mm and fever if axillary temperature was > 39.5°C. Irritability was considered of grade 3 intensity if the child cried and could not be comforted/prevented normal activity, and loss of appetite was considered grade 3 if the child did not eat at all. Grade 3 intensity for all other symptoms and AEs was defined as preventing normal everyday activity and/or causing parents/guardians to seek medical advice.

All solicited local symptoms were defined in the protocol to be considered causally related to vaccination. For all other AEs, assessment of causal relationship to vaccination was based on the investigator's clinical judgment. Use of therapeutic and prophylactic antipyretic medication was recorded within 4 days following each vaccine dose.

### Statistical Analysis

Immunogenicity analyses were performed on the according-to-protocol (ATP) immunogenicity cohort, defined as vaccinated subjects who met all eligibility criteria, complied with the protocol-defined procedures and with at least 1 antibody assay result available. To obtain 300 evaluable subjects for the ATP immunogenicity analysis (200 in the PHiD-CV group and 100 in the control group), the target sample size was 345 enrolled subjects (230 in the PHiD-CV group and 115 in the control group). ELISA geometric mean antibody concentrations (GMCs), geometric mean OPA titres (GMTs) and percentages of children reaching the predefined immunological thresholds were determined with 95% confidence intervals (95% CIs). Seropositivity/seroprotection rates and exact 95% CIs (calculated from Proc StatXact 8.1) were determined for antibodies against DTPw-HBV/Hib antigens. The safety analysis was performed on the total vaccinated cohort. Incidences of AEs were calculated with exact 95% CIs. Weight-for-age Z-scores were calculated based on the WHO Child Growth Standards using the WHO Anthro software (http://www.who.int/childgrowth/software/en).

## Results

### Study Participants

**Figure 1:**
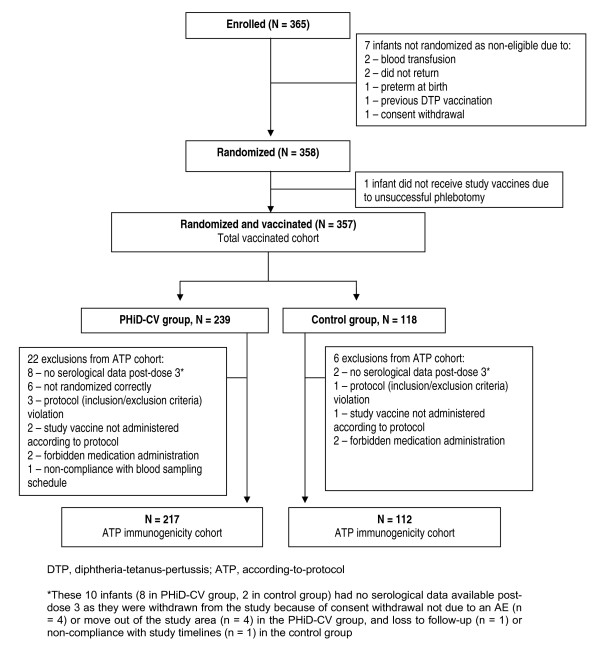
**Trial profile**.

Three hundred and sixty-five infants were enrolled of which 358 were randomized (238 in Mali and 120 in Nigeria) and 357 received at least 1 study vaccine dose (Figure 1). Subject recruitment started first in Mali and to ensure that the total target sample size would be reached, enrolment in that country continued until recruitment was also initiated in Nigeria. As a consequence, 8 more subjects than planned were enrolled and randomized in Mali. In Nigeria, 5 more subjects than planned were enrolled and randomized. There was some reluctance to withhold vaccination with PHiD-CV from these infants for whom informed consent had already been provided, since PHiD-CV had in the mean time been licensed in Europe. The study was completed by 347 infants (231 PHiD-CV, 116 control group). Reasons for withdrawal were consent withdrawal not due to an AE (n = 4) or move out of the study area (n = 4) in the PHiD-CV group, and loss to follow-up (n = 1) or non-compliance with study timelines (n = 1) in the control group.

The ATP immunogenicity cohort included 329 infants (217 PHiD-CV, 112 control group); reasons for exclusion from this cohort are given in Figure 1. The demographic characteristics of the PHiD-CV and control groups were comparable (Table 1). All infants were African. Subjects were considered to be clinically healthy at study entry according to the investigators. The mean weight-for-age Z-score was -0.50 ± 1.13 at the first vaccination visit and did not considerably change at the following visits (Table 1).

**Table 1:** Participants' characteristics (ATP immunogenicity cohort)

	PHiD-CV group (N = 217)	Control group (N = 112)	Total (N = 329)
Gender (%), female/male	50.2/49.8	50.9/49.1	50.5/49.5
Dose 1:			
mean age ± SD (weeks)	7.1 ± 1.14	7.0 ± 1.16	7.1 ± 1.14
mean weight ± SD (kg)	4.85 ± 0.76	4.74 ± 0.81	4.81 ± 0.78
mean weight-for-age Z-score ± SD	-0.45 ± 1.10	-0.61 ± 1.17	-0.50 ± 1.13
Dose 2:			
mean age ± SD (weeks)	12.2 ± 1.28	12.1 ± 1.33	12.1 ± 1.30
mean weight ± SD (kg)	5.80 ± 0.87	5.72 ± 0.93	5.78 ± 0.89
mean weight-for-age Z-score ± SD	-0.39 ± 1.14	-0.49 ± 1.22	-0.43 ± 1.17
Dose 3:			
mean age ± SD (weeks)	17.1 ± 1.25	17.0 ± 1.32	17.1 ± 1.27
mean weight ± SD (kg)	6.53 ± 0.90	6.39 ± 0.95	6.48 ± 0.92
mean weight-for-age Z-score ± SD	-0.31 ± 1.08	-0.50 ± 1.17	-0.37 ± 1.11
Mean interval between dose 3 and blood sampling visit (days)	33.4	33.3	33.4

**NOTE**. SD, standard deviation; N, number of subjects

### Immune Responses to Primary Vaccination

Before vaccination, percentages of infants with antibody concentration ≥ 0.2 μg/mL were consistent between groups for each of the vaccine pneumococcal serotypes, ranging from 14.8% to 87.9% in the PHiD-CV group and 16.1% to 84.5% in the control group (Table 2). One month post-dose 3, for each of the vaccine pneumococcal serotypes, at least 97.2% of infants in the PHiD-CV group had an antibody concentration ≥ 0.2 μg/mL, except for serotypes 6B (82.0%) and 23F (87.6%), compared to less than 10% of infants in the control group, except for serotypes 14 (35.7%) and 19F (22.5%) (Table 2). At least 93.3% of infants in the PHiD-CV group had an OPA titre ≥ 8, except for serotypes 1 (87.6%) and 6B (85.4%), compared to less than 10% in the control group, except for serotypes 7F (42.9%), 9V (24.1%) and 14 (24.5%) (Table 3). For the cross-reactive serotypes 6A and 19A, 25.8% and 43.8% of PHiD-CV recipients, respectively, had an antibody concentration ≥ 0.2 μg/mL versus less than 14% in the control group for both serotypes (Table 2) and 30.7% and 37.1% reached an OPA titre ≥ 8 versus less than 11% in the control group (Table 3). The anti-protein D antibody GMC was 44-fold higher in the PHiD-CV (3791.8 EL.U/mL [95% CI: 3448.4-4169.3]) compared to the control group (85.4 EL.U/mL [95% CI: 71.8-101.5]).

**Table 2:** IgG antibody responses (22F-ELISA) against individual pneumococcal vaccine serotypes and cross-reactive serotypes 6A and 19A (ATP immunogenicity cohort)

		PHiD-CV group			Control group		
**PHiD-CV serotypes**		**N**	**% ≥ 0.2 μg/mL (95% CI)**	**GMC, μg/mL (95% CI)**	**N**	**% ≥ 0.2 μg/mL (95% CI)**	**GMC, μg/mL (95% CI)**

1	Pre-vaccine	204	19.6 (14.4-25.7)	0.07 (0.06-0.09)	110	18.2 (11.5-26.7)	0.07 (0.06-0.09)
	Post-dose 3	217	100 (98.3-100)	2.69 (2.42-2.99)	108	1.9 (0.2-6.5)	0.03 (0.03-0.03)
4	Pre-vaccine	209	14.8 (10.3-20.4)	0.07 (0.06-0.08)	112	16.1 (9.8-24.2)	0.07 (0.06-0.08)
	Post-dose 3	217	100 (98.3-100)	3.44 (3.06-3.87)	112	2.7 (0.6-7.6)	0.03 (0.03-0.03)
5	Pre-vaccine	210	17.6 (12.7-23.5)	0.07 (0.06-0.08)	111	19.8 (12.9-28.5)	0.07 (0.05-0.08)
	Post-dose 3	217	100 (98.3-100)	4.17 (3.75-4.63)	109	3.7 (1.0-9.1)	0.03 (0.03-0.04)
6B	Pre-vaccine	205	21.0 (15.6-27.2)	0.09 (0.07-0.10)	111	31.5 (23.0-41.0)	0.10 (0.08-0.12)
	Post-dose 3	217	82.0 (76.3-86.9)	0.95 (0.76-1.20)	112	1.8 (0.2-6.3)	0.03 (0.03-0.03)
7F	Pre-vaccine	207	27.1 (21.1-33.6)	0.09 (0.08-0.11)	111	23.4 (15.9-32.4)	0.08 (0.07-0.10)
	Post-dose 3	217	99.5 (97.5-100)	3.33 (2.99-3.71)	110	1.8 (0.2-6.4)	0.03 (0.03-0.04)
9V	Pre-vaccine	208	38.5 (31.8-45.4)	0.13 (0.11-0.16)	110	39.1 (29.9-48.9)	0.15 (0.11-0.19)
	Post-dose 3	217	97.2 (94.1-99.0)	2.39 (2.06-2.76)	112	9.8 (5.0-16.9)	0.04 (0.03-0.05)
14	Pre-vaccine	206	87.9 (82.6-92.0)	0.75 (0.64-0.89)	110	84.5 (76.4-90.7)	0.76 (0.60-0.97)
	Post-dose 3	217	99.1 (96.7-99.9)	3.80 (3.24-4.46)	112	35.7 (26.9-45.3)	0.14 (0.11-0.17)
18C	Pre-vaccine	204	36.3 (29.7-43.3)	0.12 (0.10-0.15)	110	34.5 (25.7-44.2)	0.12 (0.09-0.15)
	Post-dose 3	217	99.5 (97.5-100)	10.01 (8.49-11.80)	112	3.6 (1.0-8.9)	0.03 (0.03-0.04)
19F	Pre-vaccine	208	68.3 (61.5-74.5)	0.33 (0.28-0.40)	111	68.5 (59.0-77.0)	0.36 (0.29-0.45)
	Post-dose 3	217	98.6 (96.0-99.7)	7.65 (6.55-8.93)	111	22.5 (15.1-31.4)	0.08 (0.07-0.10)
23F	Pre-vaccine	202	29.2 (23.0-36.0)	0.08 (0.07-0.10)	108	31.5 (22.9-41.1)	0.10 (0.07-0.12)
	Post-dose 3	217	87.6 (82.4-91.6)	1.10 (0.91-1.33)	112	2.7 (0.6-7.6)	0.03 (0.03-0.04)

Cross-reactive serotypes							
6A	Pre-vaccine	208	34.1 (27.7-41.0)	0.13 (0.11-0.15)	112	35.7 (26.9-45.3)	0.14 (0.11-0.18)
	Post-dose 3	217	25.8 (20.1-32.2)	0.09 (0.08-0.11)	108	7.4 (3.3-14.1)	0.04 (0.04-0.05)
19A	Pre-vaccine	205	48.3 (41.3-55.4)	0.18 (0.15-0.22)	110	44.5 (35.1-54.3)	0.20 (0.15-0.26)
	Post-dose 3	217	43.8 (37.1-50.7)	0.15 (0.13-0.18)	108	13.9 (8.0-21.9)	0.06 (0.05-0.07)

**NOTE**. GMC, geometric mean antibody concentration; pre-vaccine, before the first vaccine dose; post-dose 3, 1 month after vaccine dose 3; N, number of subjects with available results (this number varies per time point and per serotype depending on the amount of serum available for testing).

**Table 3:** OPA responses against individual pneumococcal vaccine serotypes and cross-reactive serotypes 6A and 19A 1 month after 3-dose primary vaccination (ATP immunogenicity cohort)

	PHiD-CV group			Control group		
**PHiD-CV serotypes**	**N**	**% ≥ 8 (95% CI)**	**GMT (95% CI)**	**N**	**% ≥ 8 (95% CI)**	**GMT (95% CI)**

1	105	87.6 (79.8-93.2)	83.0 (61.7-111.7)	56	5.4 (1.1-14.9)	5.0 (3.8-6.4)
4	105	100 (96.5-100)	892.5 (759.4-1049.0)	55	5.5 (1.1-15.1)	4.6 (3.9-5.5)
5	105	95.2 (89.2-98.4)	82.7 (65.4-104.4)	56	3.6 (0.4-12.3)	4.5 (3.8-5.2)
6B	103	85.4 (77.1-91.6)	538.6 (346.0-838.3)	54	9.3 (3.1-20.3)	5.7 (4.1-7.9)
7F	105	100 (96.5-100)	2733.0 (2188.3-3413.3)	49	42.9 (28.8-57.8)	31.5 (15.5-64.0)
9V	105	98.1 (93.3-99.8)	1023.7 (784.8-1335.2)	54	24.1 (13.5-37.6)	8.4 (5.8-12.4)
14	104	96.2 (90.4-98.9)	1079.2 (776.0-1500.9)	53	24.5 (13.8-38.3)	8.9 (5.7-14.1)
18C	105	98.1 (93.3-99.8)	617.6 (495.3-770.0)	56	3.6 (0.4-12.3)	4.4 (3.8-5.2)
19F	105	96.2 (90.5-99.0)	358.3 (269.9-475.5)	56	3.6 (0.4-12.3)	4.6 (3.8-5.7)
23F	104	93.3 (86.6-97.3)	881.8 (615.0-1264.4)	53	9.4 (3.1-20.7)	6.6 (4.2-10.3)

Cross-reactive serotypes						
6A	101	30.7 (21.9-40.7)	14.1 (9.4-21.2)	56	10.7 (4.0-21.9)	6.1 (4.4-8.7)
19A	105	37.1 (27.9-47.1)	11.0 (8.3-14.6)	56	5.4 (1.1-14.9)	4.3 (4.0-4.6)

**NOTE**. GMT, geometric mean titre; N, number of subjects with available results.

All children in both groups were seroprotected or seropositive for antibodies against the DTPw-HBV/Hib antigens except for 1 infant not seroprotected against PRP in the control group and 2 infants not seroprotected against HBV in the PHiD-CV group (Table 4). GMCs for anti-PRP and anti-tetanus antibodies were higher in the PHiD-CV group than in the control group (no overlap of 95% CIs), and the anti-diphtheria antibody GMC tended to be higher in the PHiD-CV group (Table 4).

**Table 4:** Immune responses against DTPw-HBV/Hib vaccine antigens 1 month after 3-dose primary vaccination (ATP immunogenicity cohort)

	PHiD-CV group			Control group		
	
Antigen (cut-off)	N	% (95% CI)	GMC (95% CI)	N	% (95% CI)	GMC (95% CI)
Diphtheria toxoid (0.1 IU/mL)	110	100 (96.7-100)	4.103 (3.527-4.773)	112	100 (96.8-100)	3.130 (2.731-3.588)
Tetanus toxoid (0.1 IU/mL)	110	100 (96.7-100)	6.484 (5.511-7.628)	112	100 (96.8-100)	4.588 (3.880-5.426)
*Bordetella pertussis *(15 EL.U/mL)	110	100 (96.7-100)	111.9 (102.0-122.7)	111	100 (96.7-100)	124.9 (111.7-139.7)
HBs (10 mIU/mL)	91	97.8 (92.3-99.7)	1835.1 (1384.0-2433.2)	96	100 (96.2-100)	1485.5 (1198.7-1840.9)
PRP (0.15 μg/mL)	110	100 (96.7-100)	18.461 (14.256-23.907)	112	99.1 (95.1-100)	10.137 (7.515-13.673)

**NOTE**. GMC, geometric mean antibody concentration; HBs, hepatitis B surface antigen; PRP, *Haemophilus influenzae *type b polyribosylribitol phosphate; N, number of subjects with available results

### Reactogenicity and Safety

**Figure 2:**
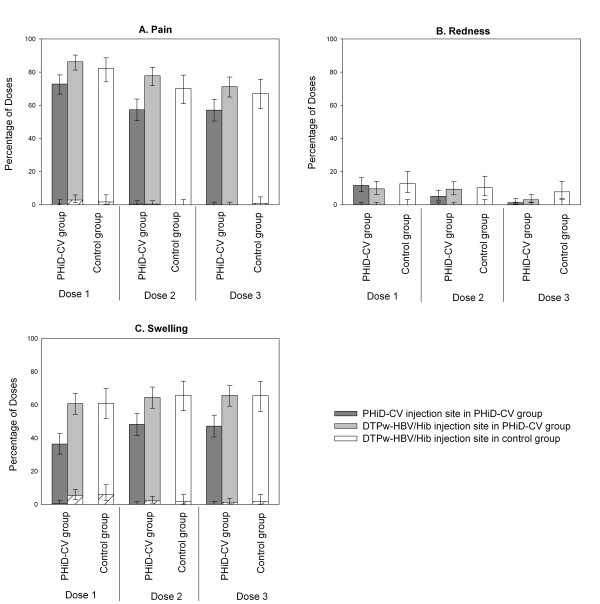
**Local symptoms**. Percentage of doses followed by pain (A), redness (B) and swelling (C) at the specified injection site of any intensity and grade 3 intensity (hatched areas) (ATP immunogenicity cohort). Error bars indicate 95% CIs. Grade 3 pain: crying when limb was moved/spontaneously painful; grade 3 redness/swelling: diameter > 30 mm.

Overall incidences of solicited local events during the 4-day post-vaccination period were similar between the PHiD-CV and control groups (pain: 80.8% [95% CI: 77.7%-83.6%] versus 73.2% [95% CI: 68.3%-77.8%]; redness: 10.9% [95% CI: 8.7%-13.4%] versus 10.3% [95% CI: 7.3%-13.9%]; swelling: 65.3% [95% CI: 61.7%-68.9%] versus 64.1% [95% CI: 58.8%-69.1%]). In both groups, pain was the most frequently reported solicited local symptom (Figure 2). In the PHiD-CV group, pain and swelling were reported more frequently at the DTPw-HBV/Hib than at the PHiD-CV injection site (Figure 2). Overall incidences of solicited general events were similar between the PHiD-CV and control groups (drowsiness: 3.8% [95% CI: 2.5%-5.5%] versus 3.7% [95% CI: 2.0%-6.3%]; fever: 54.9% [95% CI: 51.1%-58.6%] versus 57.0% [95% CI: 51.6%-62.2%]; irritability 47.9% [95% CI: 44.2%-51.7%] versus 43.0% [95% CI: 37.8%-48.4%]; loss of appetite 5.5% [95% CI: 4.0%-7.5%] versus 4.8% [2.8%-7.6%]). Fever (axillary temperature ≥ 37.5°C) was the most commonly reported solicited general event in both groups, with only 1 report of grade 3 fever (PHiD-CV group, dose 3). Antipyretic medication use within 4 days post-vaccination was frequent and similar in both groups (for 88.7% [95% CI: 86.1%-90.9%] of doses in the PHiD-CV group and 87.2% [95% CI: 83.2%-90.5%] in the control group). Antipyretic medication was taken prophylactically for 9.6% (95% CI: 7.5%-12.0%) of the vaccine doses in the PHiD-CV group and 8.5% (95% CI: 5.8%-12.0%) in the control group.

**Figure 3:**
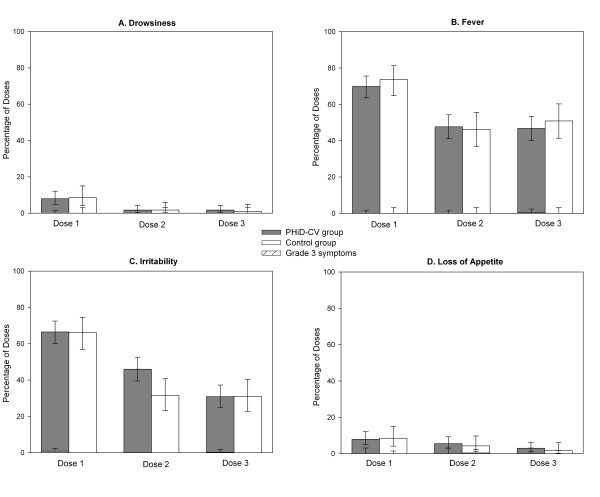
**General symptoms**. Percentage of doses followed by drowsiness (A), fever (B), irritability (C) and loss of appetite (D) of any intensity and grade 3 intensity (hatched areas) (ATP immunogenicity cohort). Error bars indicate 95% CIs. Grade 3 drowsiness: prevented normal activity; grade 3 fever: axillary temperature > 39.5°C; grade 3 irritability: crying that could not be comforted/prevented normal activity; grade 3 loss of appetite: child did not eat at all.

The incidences of grade 3 solicited general symptoms (Figure 3) and grade 3 pain and redness (Figure 2) following each vaccine dose were low (≤2.9%) in both groups. The incidence of grade 3 swelling at the PHiD-CV injection site was also low (≤0.4%) while grade 3 swelling at the DTPw-HBV/Hib injection site was 5.4% and 5.9% in PHiD-CV and control group respectively after the first dose and ≤2.1% after subsequent doses (Figure 2).

The percentage of doses followed by at least 1 unsolicited AE was similar in both groups (following 53.0% [95% CI: 49.3%-56.8%] of doses in the PHiD-CV group and 57.3% [95% CI: 51.9%-62.5%] in the control group). The most common unsolicited AEs were gastroenteritis (9.8% [95% CI: 7.7%-12.2%] in PHiD-CV group, 10.3% [95% CI: 7.3%-13.9%] in control group), rhinitis (16.0% [95% CI: 13.4%-18.9%] and 14.8% [95% CI: 11.3%-19.0%]) and rhinobronchitis (15.0% [95% CI: 12.4%-17.8%] and 16.2% [95% CI: 12.5%-20.5%]). Unsolicited AEs with a causal relationship to vaccination were reported following 5.7% (95% CI: 4.1%-7.6%) of doses in the PHiD-CV group and 9.7% (95% CI: 6.8%-13.3%] of doses in the control group (mainly injection site induration; following 4.1% and 6.8% of doses, respectively). The incidence of grade 3 unsolicited AEs was low, following 1.1% of doses in both groups, and none was considered to be causally related to vaccination.

Seven SAEs were reported in 5 children in the PHiD-CV group (4 cases of bronchopneumonia, 1 case of malaria, 1 case of febrile convulsions and 1 head injury [haematoma] secondary to a car accident) and none in the control group. None of the SAEs were considered to be causally related to vaccination and all infants recovered without sequelae.

## Discussion

Pneumococcal conjugate vaccines, including PHiD-CV, are being introduced in African countries under the Advance Market Commitments for pneumococcal disease piloted by GAVI [2]. In our study, PHiD-CV was immunogenic for all vaccine pneumococcal serotypes and NTHi protein D when given as a 3-dose primary vaccination course at 6, 10 and 14 weeks of age and co-administered with DTPw-HBV/Hib and OPV to Malian and Nigerian infants. Vaccine tolerability was comparable between the group administered PHiD-CV and the control group administered DTPw-HBV/Hib and OPV only.

One month after PHiD-CV primary vaccination at 6, 10 and 14 weeks of age, for each of the 10 vaccine serotypes high percentages of infants had an antibody concentration ≥ 0.2 μg/mL and an OPA titre ≥ 8. For several serotypes, this was despite high percentages of infants with pre-vaccination antibody concentration ≥ 0.2 μg/mL. These immune responses are consistent with previous primary vaccination studies in which PHiD-CV was administered using the same schedule [15] or at 2, 4 and 6 months of age [15,20-22]. As in other pneumococcal conjugate vaccine studies [8,14,29-33], serotype 6B was one of the low immunogenic serotypes. However, pneumococcal conjugate vaccine trials have reported high efficacy against IPD or acute otitis media even for serotypes with lower antibody responses [8,14,29,30,34]. The high proportions of infants with a pre-vaccination antibody concentration ≥ 0.2 μg/mL we observed in this study for several vaccine serotypes can probably be explained by maternal antibodies transferred during the last months of pregnancy rather than early exposure to *S. pneumoniae*, given that antibody concentrations and percentages of infants with antibody concentration ≥ 0.2 μg/mL significantly declined in the control group by the time of post-vaccination blood draw at approximately 5 months of age. Our data however indicate that the presence of these high levels of maternal antibodies did not hamper immune responses to early infant PHiD-CV primary vaccination. It is well known that pneumococcal exposure and acquisition of pneumococcal nasopharyngeal carriage may start very early in sub-Saharan infant populations [35,36] and recent publications have indicated that pneumococcal colonization at the time of primary vaccination could possibly have a negative impact on infant pneumococcal conjugate vaccine responses [37-39]. Our results therefore seem to support the need to maintain early vaccination with pneumococcal conjugate vaccines in this population.

Anti-pneumococcal immune responses in this population of infants tended to be higher than those observed in European PHiD-CV primary vaccination studies [15,18,20]. A PHiD-CV study conducted in Chile also reported stronger immune responses for all serotypes compared to European data [21] and a study of PHiD-CV conducted in the Philippines and Poland found greater immunogenicity in Filipino infants [15] despite the use of an accelerated schedule compared to the more immunogenic 2, 4 and 6 months schedule in Poland. Genetic factors, early exposure to pneumococcal serotypes or nasopharyngeal carriage of pneumococcal serotypes might have contributed to this effect [40].

Good immune responses were also observed against DTPw-HBV/Hib, which was consistent with another study of PHiD-CV co-administered with DTPw-HBV/Hib [15,28], and proportions of infants who were seroprotected or seropositive for antibodies against the DTPw-HBV/Hib antigens were high (≥ 97.8%). Antibody concentrations against the PRP, tetanus and diphtheria antigens were higher when PHiD-CV was co-administered, presumably due to enhancement of these immune responses by the tetanus toxoid and diphtheria toxoid carrier proteins used for serotypes 18C and 19F, respectively, in PHiD-CV [28]. High antibody GMCs and seroprotection rates were however observed for these antigens in both groups, including the PRP antigen, confirming the good immunogenicity of a reduced PRP content Hib vaccine (2.5 μg of PRP) compared to the more usual 10 μg PRP vaccines [41-45].

Addition of PHiD-CV to the routine EPI vaccination schedule at 6, 10 and 14 weeks of age did not result in increased incidences of AEs (solicited or unsolicited) relative to DTPw-HBV/Hib and OPV administered alone in the control group. There were no SAEs considered causally related to the vaccines. The overall incidences of solicited symptoms were similar between the 2 groups, with pain and fever being the most frequently reported local and general symptoms. The incidences of pain and swelling were higher at the DTPw-HBV/Hib injection site than at the PHiD-CV injection site, which was in line with another study of PHiD-CV or 7vCRM co-administered with a DTPw-based vaccine [15,16]. Prophylactic use of antipyretics is recommended when PHiD-CV is co-administered with whole-cell pertussis containing vaccines [46]. In our study, antipyretic medication use at the time of vaccination was common in both groups (for 87-89% of the doses) and antipyretic prophylaxis was used in 8-10% of vaccine doses, with similar prophylactic antipyretic use after each dose (data not shown). The incidences of grade 3 solicited symptoms were low, apart from the incidence of grade 3 swelling at the DTPw-HBV/Hib injection site after the first vaccine dose. The overall incidence of unsolicited AEs was also comparable between groups and the most frequent unsolicited AEs reported were in line with events expected in the paediatric age group studied.

## Conclusion

PHiD-CV was immunogenic for all vaccine pneumococcal serotypes and NTHi protein D in Malian and Nigerian infants when given as a 3-dose primary vaccination course according to the routine EPI vaccination schedule at 6, 10 and 14 weeks of age and co-administered with DTPw-HBV/Hib and OPV. Tolerability was generally comparable between the PHiD-CV and control vaccination groups and pain and swelling were less frequent at the PHiD-CV injection site compared to the DTPw-HBV/Hib injection site. These data provide valuable information for PHiD-CV paediatric use in sub-Saharan African settings.

## List of abbreviations

7vCRM: 7-valent pneumococcal CRM_197 _conjugate vaccine; AE: adverse event; ATP: according-to-protocol; CI: confidence interval; DTPw-HBV/Hib: diphtheria-tetanus-whole-cell pertussis-hepatitis B/*Haemophilus influenzae *type b vaccine; EL.U: ELISA unit; ELISA: enzyme-linked immunosorbent assay; EPI: Expanded Program on Immunization; GAVI: Global Alliance for Vaccines and Immunization; GMC: geometric mean concentration; GMT: geometric mean titre; GSK: GlaxoSmithKline; HBs: hepatitis B surface antigen; IPD: invasive pneumococcal disease; NTHi: non-typeable *Haemophilus influenzae*; OPA: opsonophagocytic activity; OPV: oral live attenuated poliovirus vaccine; PHiD-CV: pneumococcal non-typeable *Haemophilus influenzae *protein D conjugate vaccine; PRP: polyribosylribitol phosphate; SAE: serious adverse event; WHO: World Health Organization.

## Competing interests

AlDi, AID, GS, AB and AmDo declare having received grants from GlaxoSmithKline (GSK) Biologicals for study logistics and patient recruitment/follow-up for the reported study; OOO, AmDi, OAK and YAK declare having received honoraria and/or consulting fees from GSK Biologicals. OOO also has board membership with GSK Biologicals and AlDi received travel fees for presenting the study results at conferences. NF, DB, JPY, MM and LS are employed by GSK Biologicals and JPY, DB, MM and LS own stocks.

## Authors' contributions

AlDi, OOO and YAK helped design and plan the reported study, collected data and provided interpretation of the results; GS and AB collected data; AID, AmDi, and AmDo helped design and plan the reported study and collected data; OAK collected data and helped interpret the results; NF helped design the study and did the statistical analyses; JPY, MM and LS designed and planned the study and helped analyze and interpret the results. DB helped analyze and interpret the results. All authors critically reviewed the different drafts of the manuscript and approved the final version.

## Pre-publication history

The pre-publication history for this paper can be accessed here:

http://www.biomedcentral.com/1471-2458/11/882/prepub
